#  Retraction of: Virtual reality training for radiation safety in cardiac catheterization laboratories – an integrated study

**DOI:** 10.1093/rpd/ncag084

**Published:** 2026-07-22

This is a retraction of Asahi Fujiwara, Sota Fujimoto, Ren Ishikawa, Aoi Tanaka, Virtual reality training for radiation safety in cardiac catheterization laboratories – an integrated study, *Radiation Protection Dosimetry*, Volume 200, Issue 15, September 2024, Pages 1462–1469, https://doi.org/10.1093/rpd/ncae187

In December 2024, the journal was notified by a reader of concerns regarding the validity of the ethical approval for the research presented and the data underlying the results and conclusions presented in the article. The journal requested that the authors provide the ethical approval documentation, and the raw data associated with the study. Despite repeated attempts to contact all authors over an extended period, the journal did not receive satisfactory responses or documentation addressing these concerns. In the absence of verifiable ethical approval and supporting data, the Editor in Chief no longer has confidence in the integrity of the study’s findings. For this reason, and in accordance with COPE Retraction Guidelines, the article is hereby retracted. The authors have been informed of this retraction.

